# Exosomes Derived from Hypoxic Colorectal Cancer Cells Transfer miR-410-3p to Regulate Tumor Progression

**DOI:** 10.7150/jca.33232

**Published:** 2020-05-25

**Authors:** Xiufeng Hu, Yu Mu, Jie Liu, Xiaoqian Mu, Fangfang Gao, Lijuan Chen, Huijuan Wu, Hongbo Wu, Wenjing Liu, Yanqiu Zhao

**Affiliations:** Department of Internal Medicine, Affiliated Cancer Hospital of Zhengzhou University, Henan Cancer Hospital, Zhengzhou, China.

**Keywords:** colorectal cancer, hypoxia, exosome, progression, miR-410-3p, PTEN

## Abstract

Hypoxia is a common characteristic of solid tumors and is associated with cancer progression and poor outcomes. However, the roles and specific mechanisms of exosomes and hypoxia during cancer progression still remain unclear. Herein, we found that exosomes secreted from hypoxic colorectal cancer (CRC) cells promoted the proliferation, migration, invasion, and metastasis of normoxic CRC cells, and these hypoxic exosomes exerted their biological effects depending on miR-410-3p. We discovered that miR-410-3p was highly enriched in hypoxic CRC-derived exosomes in a HIF1α or HIF2α-dependent manner, and miR-410-3p levels positively associated with poor prognosis of CRC. Moreover, decreased PTEN levels caused by hypoxic CRC cells-derived exosomal miR-410-3p increased activation of PI3K/Akt as well as tumor progression. Conversely, inhibition of miR-410-3p or PI3K/Akt signaling pathway effectively decreased hypoxic CRC cells-derived exosomes-mediated tumor progression. In conclusion, our findings indicate that the hypoxic microenvironment in CRC may promote tumor cells to release miR-410-3p-rich exosomes that are transferred to normoxic cells to enhance tumor progression, revealing a new investigation into the therapeutic targets of exosome for CRC treatment.

## Introduction

Colorectal cancer (CRC) is one of the most prevalent carcinomas and the second leading cause of cancer-related death worldwide 1. Although standard surgical resection and adjuvant treatments such as target therapy and chemotherapy are widely applied in treatment of CRC, the poor prognosis of CRC was caused by early metastasis, aggressiveness and poor response to available treatments 2. Hence, an in-depth understanding of the specific molecular mechanisms of CRC metastasis and the discovery of new prognostic and diagnostic related markers are critical.

Recently, the tumor microenvironment has been shown to play a critical role in immunosuppression and tumor metastasis 3,4. Hypoxia and infiltrated inflammatory cells are two major factors which are closely related to the tumor microenvironment 5. The changes of oxygen levels which the oxygen pressure is under 5mmHg leading to hypoxia regions, can mediate CRC resistance and progression in the early stages 6. The effect of hypoxia-induced CRC metastasis has been shown to mediate epithelial-mesenchymal transition (EMT) of cancer cells and during EMT, epithelial cells transform into mesenchymal cells with strong ability to invade and metastasis 7. However, the concrete mechanism by which hypoxia induces the progression of CRC remains largely unknown.

A large number of previous studies have shown that in addition to secreting soluble mediators such as cytokines, chemokine and growth factors, the main mediator of intercellular communication in the tumor microenvironment can also transmit exosomes which could carry proteins, lipids, mRNAs, and MicroRNA (miRNA) 8,9. MiRNA delivered by exosomes is especially interesting, because miRNAs could regulate a range of target genes in recipient cells, mediating a series of pathophysiological responses 10. MiRNA in exosomes derived from tumor cells have been demonstrated to support cancer growth by remodeling the tumor microenvironment 11-14. For example, exosomal miR-21 derived by cancer cells increased M2-like polarization of tumor-associated macrophages in head and neck squamous cell carcinoma 15. Noteworthy, emerging evidence suggests that the hypoxic microenvironment is closely related to miRNA changes in exosomes secreted by tumor cells 16. In a previous study, hypoxic pancreatic cancer-derived exosomal miR-301a facilitated the lung metastasis via inducing M2 macrophage 17. In addition, hypoxia promoted exosomal miR-103a secretion in lung cancer cells, thereby enhancing tumor angiogenesis and vascular permeability, ultimately leading to tumor metastasis 18. However, the roles and mechanisms underlying the relationship between exosomal miRNA changes and hypoxia during CRC progression remain unclear.

Given the crucial roles of hypoxia, exosome and miRNA in dictating tumor progression and metastasis, we speculated that hypoxia can promote CRC progression by inducing tumor cells to secrete specific types of exosomal miRNAs. In the present study, we have investigated that CRC cells-secreted exosomal miR-410-3p in hypoxic microenvironment promotes progression and metastatic potential of normoxic CRC cells via PTEN/PI3K/Akt pathway. Thus, the data show that miR-410-3p is a potential target for exosome-mediated tumor progression.

## Materials and Method

### Patient samples

Primary CRC tissue samples were obtained from 60 patients who underwent curative resection at Affiliated Tumor Hospital of Zhengzhou University. All included patients were identified as adenocarcinoma of colorectal by histopathology. Moreover, all patients were devoid of neoadjuvant chemotherapy or radiotherapy before surgical resection. All samples were collected with informed consent from patients, and all related procedures were performed with the approval of the internal review and ethics boards of Affiliated Tumor Hospital of Zhengzhou University. Table 1 shows the clinicopathologic features and tissues of the patients.

### Cell culture and hypoxia treatment

The human CRC cell lines (DLD-1 and HT29) were purchased from the Chinese Academy of Sciences in Shanghai. Cells were cultured in RPMI 1640 medium (Gibco, USA) with 10% fetal bovine serum (FBS) (Gibco, USA) at 37°C in a humidified atmosphere with 5% CO_2_ containing 20% O_2_. For hypoxic conditions, DLD-1 and HT29 cells were cultured in a hypoxia cell incubator of 1% O_2_ as previously shown 17.

Phosphoinositide 3-kinase (PI3K) inhibitor LY294002 (Calbiochem, Nottingham, UK) were used at a concentration (1 μM). Dimethyl sulfoxide (DMSO) was purchased from Sigma-Aldrich (St. Louis, USA). GW4869 was purchased from MedChemExpress (USA).

### Plasmid constructs, miRNAs, and transfections

The PTEN plasmid vector were chemically synthesized, constructed, sequenced and identified by Shanghai GeneChem Chemical Technology, Co. Ltd, China. Vectors of HIF-1α-siRNA, HIF-2α-siRNA, or negative control RNA (NC) were also chemically synthesized, constructed, sequenced and identified by Shanghai GeneChem Chemical Technology, Co. Ltd, China. CRC cells were transfected with siRNAs or NC RNA using Xtreme GENE siRNA Transfection Reagent (Roche, USA) according to the manufacturer's instructions. The miR-410-3p-knockout lentiviruses, and control lentivirus were also chemically synthesized, constructed, sequenced and identified by Shanghai GeneChem Chemical Technology, Co. Ltd, China. Forty-eight hours after transfection, cells were plated for a functional assay or harvested for RNA and protein analyses. miR-410-3p mimics and inhibitor were obtained from RiboBio Co. Ltd, China. The RNA was transfected using Lipofectamine 2000 (Invitrogen, USA), following the manufacturer's instructions.

### Exosomes Isolation and Uptake

The DLD-1 and HT29 cells were cultured in the culture medium with 10% FBS until 70%-80% confluent; afterwards, medium was replaced with 10% exosome-depleted FBS and cultured under normoxic or hypoxic (1% O_2_) conditions. Then, the cell culture medium was harvested after 2 days (50 mL) and ultra-centrifuged as previously shown 19. In brief, culture medium was centrifuged at 2000×g for 15min and 10,000×g for 40min to remove cell debris, and then subjected to ultracentrifugation at 100,000×g for 90 min with Hitachi ultracentrifuge CP100NX. The pelleted exosomes were resuspended in PBS and stored at -80°C until further use. Freshly isolated exosomes were analyzed using nanoparticle tracking analysis (NTA) measurements and size distribution was analyzed with NanoSight LM10 system (NanoSight).

Purified exosomes were labeled with PKH67 Green Fluorescent Cell Linker Kit (Sigma-Aldrich) according to manufacturer's instructions. Then, the labeled exosomes were resuspended and added to the DLD-1 and HT29 cells for exosomes uptake studies. After incubation for 2 hours at 37°C, cells were observed by fluorescence microscopy.

### Electron microscopy

Exosomes were isolated and loaded on to a carbon-coated electron microscopy grid. The samples were employed to be examined by transmission electron microscopy as previously described 20.

### RNA isolation and quantitative real-time PCR (qRT-PCR)

The total RNA from CRC cells was isolated using the Trizol Reagent (Invitrogen, USA) according to the manufacturer's instructions. After detection of RNA concentration, 1μg of total RNA was reverse transcribed into cDNA using the PrimeScript™ RT reagent kit (Toyobo, Osaka). cDNA was used for subsequent qRT-PCR using the SYBR-Green PCR Master Mix (Takara, Osaka). Each reaction was run on the BioRad IQ5 Real time PCR machine (BioRad, USA). Relative expression was calculated using the 2^-ΔΔCt^ method. The sequences of primers used in the study are shown in **Supplementary Table 1**.

### Luciferase reporter assay

For miRNA target report assays, the 3′-UTR sequences of PTEN, and miRNA binding sites were amplified from the genomic DNA and sub-cloned into the psi-CHECK2 (Promega, USA). For the miRNA target reporter assay, HEK293T were co-transfected with psi-CHECK-2 vectors and miRNA mimics or negative control using Lipofectamine 2000. Renilla luciferase reporter vector pRL-SV40 (Promega, USA) was provided as an internal transfection control. The total cell lysates were harvested 48h after transfection, and luciferase activities were determined using Dual-Luciferase reporter system (Promega, USA) according to the manufacturer's instructions.

### Western blot

Cells were lysed using a RIPA buffer, including a protease inhibitor cocktail (Thermo Scientific, USA). The proteins were separated by SDS-PAGE gels and transferred to PVDF membranes (Millipore, USA). After blocking with 5% non-fat milk, the membranes were incubated with primary antibodies at 4°C overnight. The HRP-conjugated secondary antibodies were used to incubate the membranes for 2h at room temperature. The membranes were washed and incubated for 1h at room temperature with HRP-conjugated secondary antibodies. Proteins were detected using a Bio-Rad ChemiDoc XRS+System. Bio-Rad Image Lab software was used for densitometric analysis. The following primary antibodies were purchased: anti-CD81 (1:2000; Cell Signaling, USA), anti-TSG101 (1:1000; Proteintech, USA), anti-CD9 (1:1000; Abcam, USA), anti-CD63 (1:1000; Abcam, USA), anti-PTEN (1:1000; Cell Signaling, USA), anti-PI3K p85 (1:1000, Abacm, USA), the anti-phospho-PI3K p85 (1:1000, Cell Signaling, USA), anti-p-AKT (phosphor S473) (1:1000; Abcam, USA), anti-AKT (1:1000; Abcam, USA), anti-GAPDH (1:5000; Santa Cruz, CA).

### Colony formation and wound healing assay

For colony formation detection, 500 cells were planted in 6-well plates and cultured for 2 weeks. Cells were then fixed with 4% paraformaldehyde and stained with 0.5% crystal violet. The assay was performed three times for each treatment.

Cells were grown to 80-90% confluence in 24-well plates, and a wound was made by dragging a plastic pipette tip across the cell surface. The remaining cells were washed three times in PBS to remove cellular debris and incubated at 37°C with serum-free medium. Migrating cells at the wound front were photographed after 24h. All experiments were performed in triplicate. The area of the wound was measured with Image J software (NIH, USA).

### Transwell invasion assay

Transwell invasion assays were performed using 24-well Transwells (8 μm pore size; Corning, USA) uncoated with Matrigel. Cell invasion assays were performed using 24-well Transwells (8µm pore size; Corning, USA) pre-coated with Matrigel (Falcon 354480; BD Biosciences, USA). In total, 1×10^5^ cells were suspended in 500 μl RPMI 1640 containing 1% FBS and added to the upper chamber, while 750 μl RPMI 1640 containing 10% FBS was placed in the lower chamber. After 48h of incubation, Matrigel and the cells remaining in the upper chamber were removed using cotton swabs. Cells on the lower surface of the membrane were fixed in 4% paraformaldehyde and stained with 0.5% crystal violet. Cells in 5 microscopic fields (at ×200 magnification) were counted and photographed. All experiments were performed in triplicate.

### Animal experiments

All animal experiments were performed according to our institutions' guidelines for the use of laboratory animals and were approved by the Institutional Animal Care and ethical committee of Affiliated Tumor Hospital of Zhengzhou University. For the liver and lung metastasis experiment, the 6-8 weeks old nude mice were divided into six randomized groups (n=6 per group), and HT29 cells (5 × 10^5^) alone or HT29 cells (5 × 10^5^) treated with hypoxic (with or without GW4869) or normoxic exosomes or HT29 cells (5 × 10^5^) transfected with miR-410-3p mimics/NC in 100 μl were injected into the mice via tail vein. Thirty days after cells injection, the mice were euthanized and were necropsied to assess metastatic burden. The liver and lung tissues of mice were further examined by H&E, IHC staining.

### Statistical analysis

All data are presented as mean ± SD. (N ≥ 3). The comparisons of groups were analyzed by Student* t* test or one-way ANOVA. Overall survival (OS) were analyzed by Kaplan-Meier analysis with log-rank tests. All statistical analyses were performed using the SPSS 23.0. A value of *P* < 0.05 was considered statistically significant.

## Results

### Exosomes derived from hypoxic CRC cells increase the proliferation, migration, and invasion of normoxic CRC cells

**Figure 1:**
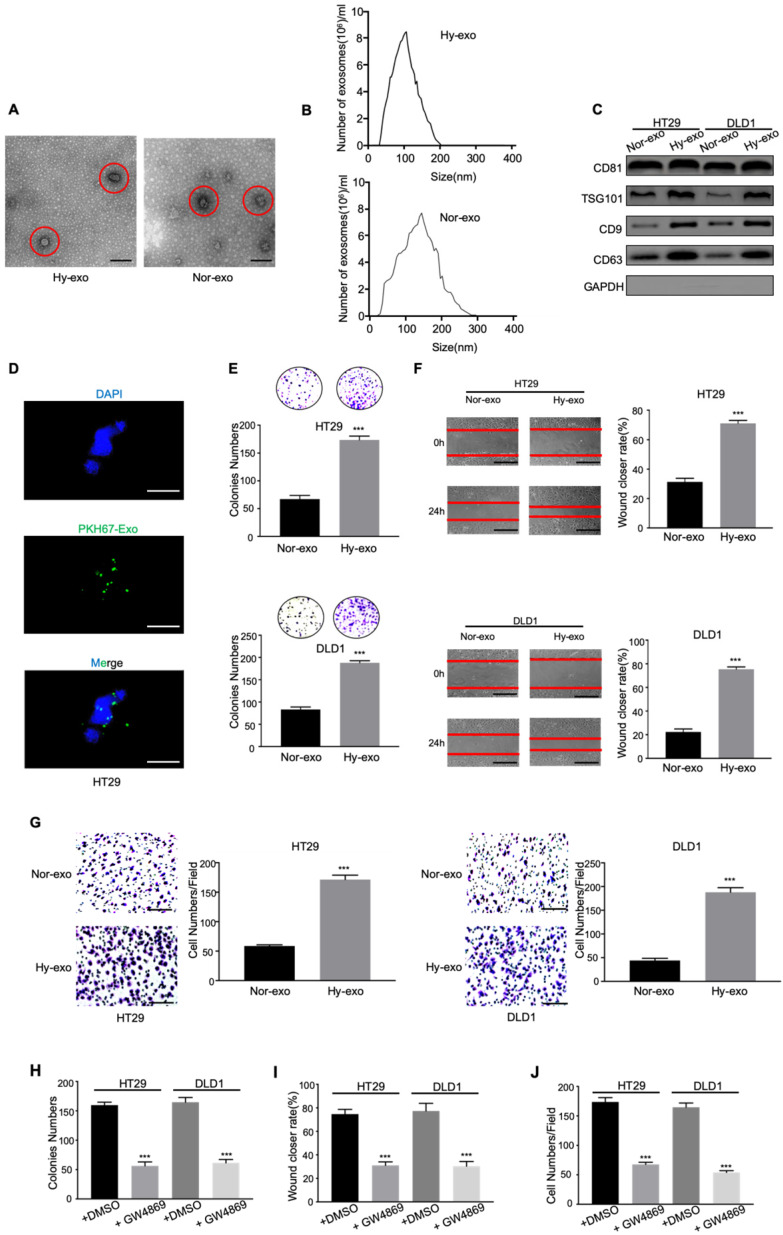
** Exosomes derived from hypoxic CRC cells increase the proliferation, migration, and invasion of normoxic CRC cells.** (**A**) Electron micrograph of exosomes isolated from HT29 exosome-free medium under normal or hypoxia revealing the typical morphology and size. (**B**) NTA of HT29-Hy-exo or HT29-Nor-exo isolated by ultracentrifugation. (**C**) Western blot analysis showing the presence of CD81, TSG101, CD9, and CD63 in exosomes derived from normoxic or hypoxic HT29 and DLD1 cells. (**D**) Representative immunofluorescence image shows the internalization of PKH67-labeled HT29-derived exosomes (green) under hypoxia by normoxic HT29 cells. (**E**) (**F**), and (**G**) Cell proliferation, migration and invasion capacity of CRC cells (DLD1 and HT29) treated with normoxic or hypoxic exosomes was determined by the colony formation, wound healing assay and transwell invasion assay, respectively. (**H**) (**I**), and (**J**) Cell proliferation, migration and invasion capacity of CRC cells (DLD1 and HT29) treated with exosomes isolated from hypoxic medium with or without GW4869 was determined by the colony formation, wound healing assay and transwell invasion assay, respectively. Representative photographs of migratory or invaded cells (magnification, ×200) are shown; Error bars, SD. ***P < 0.001.

Tumor-derived exosomes were initially isolated from the conditioned media of CRC cells (DLD1 and HT29) cultured under normoxia and hypoxia (1% O_2_) after 48 hours. The morphology of the exosomes was observed via electron microscopy. As shown in Figure 1A and 1B, electron microscopy showed that typical rounded particles ranged from 30-150 nm, and NTA exhibited a similar size distribution of exosomes. Western blotting analysis revealed that the exosomes were enriched with the exosomal markers CD81, TSG101, CD9 and CD63 (Figure 1C), which was proven the effective isolation of the exosomes. In addition, we labeled the exosomes with fluorescent PKH67 and confirmed that the labeled exosomes were taken up by HT29 cells during 2h coculture system as measured by fluorescence microscopy (Figure 1D).

To determine the effects of hypoxic CRC cell-derived exosomes on normoxic CRC cells, we analysed the proliferation, migration and invasion abilities of CRC cells treated with exosomes derived from hypoxic CRC cells. As shown in Figure 1E, hypoxic CRC cell-derived exosomes significantly increased the proliferation compared with normoxic CRC cell-derived exosomes. Wound healing assay showed that hypoxic exosomes derived from both CRC cell lines significantly promoted CRC cells migration compared with control (Figure 1F). The result was confirmed by transwell assay (Figure 1G). To further determine the pro-metastatic effect of exosomes derived from hypoxic CRC cells *in vitro*, we added GW4869, an exosome secretion inhibitor, to hypoxic CRC cells culture system to assess this effect. Compared with the addition of DMSO, the addition of GW4869 inhibited cell viability, migration, and invasion in CRC cells as expected (Figure 1H-J). These results demonstrate that hypoxic CRC cell-derived exosomes promoted the proliferation, migration and invasion of CRC cells.

### miR-410-3p is highly expressed in exosomes secreted from hypoxic CRC cells and can be transferred through the exosomes

**Figure 2:**
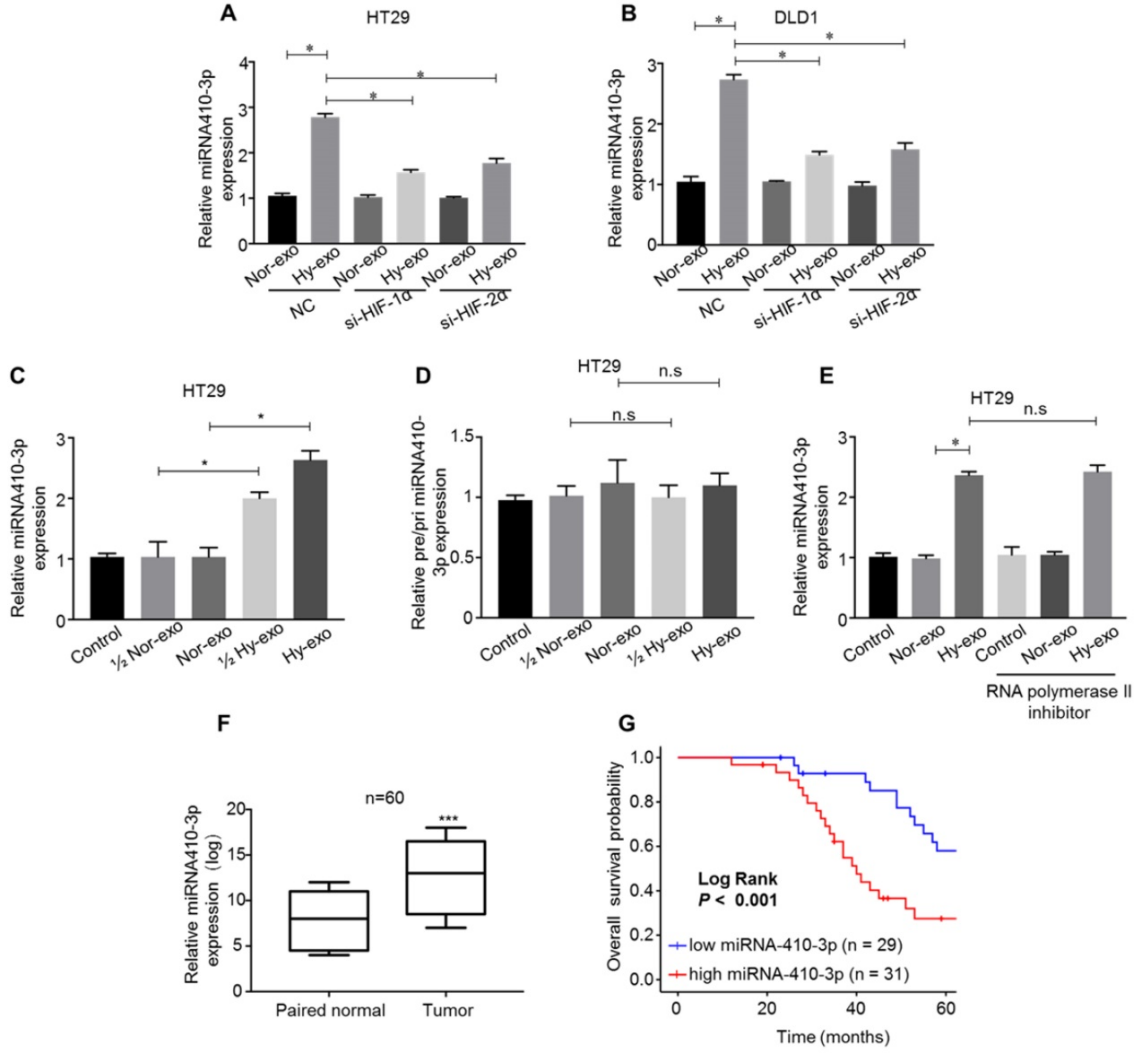
** miR-410-3p is highly expressed in exosomes secreted from hypoxic CRC cells and can be transferred through the exosomes**. (**A-B**) After transfection with HIF-1α siRNA or HIF-2α siRNA in CRC cells, miR-410-3p expression in exosomes secreted from hypoxic or normoxic CRC cells after a 12h treatment was assessed by quantitative real-time PCR. (**C-D**) The level of pre/pri-miR-410-3p and mature miR-410-3p in normoxic HT29 receiving exosomes after a 12h treatment. The level of pre/pri- and mature miR-410-3p was assessed by quantitative real-time PCR. (**E**) RNA polymerase II inhibitor did not affect the increase of miR-410-3p in recipient HT29 cells. HT29 cells were treated with polymerase II inhibitors (5,6-dichloro-1-b-D-ribofuranosylbenzimidazole, 20 mM) for 3 hr and then incubated with either normoxic or hypoxic HT29-derived exosomes. Levels of mature miR-410-3p were assessed by quantitative real-time PCR. (**F**) The expression levels of miR-410-3p in 60 pairs of CRC and normal tissues by quantitative real-time PCR analyses. (**G**) Kaplan-Meier analysis with a log-rank test for OS in 60 CRC patients according to miR-410-3p expression. Error bars, SD. ns, not significant; *P<0.05; ***P<0.001.

Recently, miRNAs have been wrapped in exosomes, and play a critical role in CRC chemoresistance and progression 21. Growing evidence has shown that miR-410-3p acts as an oncogene associated with tumor progression in various types of cancer, including colorectal cancer 22, pancreatic ductal adenocarcinoma 23 and breast cancer 24. Firstly, we detected the expression of miR-410-3p, and the results showed that levels of miR-410-3p were highly expressed in the hypoxic CRC cells-secreted exosomes compared with normoxic CRC cells-secreted exosomes (Figure 2A and B). To study whether hypoxia-induced miR-410-3p expression depends on HIF-1α or HIF-2α, the expression of HIF-1α or HIF-2α in CRC cells was knocked down by small interfering RNA. In normoxic conditions, exosomal miR-410-3p expression levels were not significantly affected by either HIF-1α or HIF-2α knockdown. In hypoxic conditions, knockdown HIF-1α or HIF-2α expression significantly decreased exosomal miR-410-3p expression (Figure. 2A and B). These results suggest that exosomal miR-410-3p expression under hypoxia was dependent on both HIF-1α and HIF-2α. Given the crucial roles of miR-410-3p, exosome and hypoxic tumour microenvironment in dictating CRC progression, we speculated that the crosstalk between hypoxic tumour microenvironment and tumor cells could promote tumor metastasis through hypoxic exosome-mediated transfer of miR-410-3p. Therefore, we chose miR-410-3p for further research. We clarified that hypoxic CRC cells-secreted miR-410-3p can be transferred to normoxic CRC cells by exosomes and then detected the miR-410-3p levels in CRC cells treated with exosomes secreted from CRC cells under hypoxic and normoxic conditions. An elevated level of mature miR-410-3p in a dose dependent manner was observed in HT29 cells after treatment with hypoxia-derived exosomes (Figure 2C). Likewise, pri- or pre-miR-410-3p was also no significantly difference in the normoxic or hypoxic CRC cells-secreted EVs (Figure 2D). Besides, treatment of RNA polymerase II inhibitor did not influence the increase of miR-410-3p in CRC cells treated with exosomes secreted from CRC cells under hypoxic conditions, showing that increase of miR-410-3p arose from exosomes-mediated miRNA transfer, but not endogenous miR-410-3p induction (Figure 2E).

As we observed miR-410-3p was substantially upregulated in exosomes secreted from hypoxic CRC cells compared to normoxic cells, we believed it was critical to establish clinical relevance in human CRC. We assessed the level of miR-410-3p in 60 pairs of CRC and normal tissues obtained from Affiliated Tumor Hospital of Zhengzhou University and found that the expression was significantly higher in the tumor than that in the paired normal tissues (Figure 2F). Next, Kaplan-Meier analysis with the log-rank test on the overall survival (OS) of these 60 CRC patients, showed that high miR-410-3p predicted a poor OS in CRC patients (Figure 2G).

### Hypoxic CRC cells-secreted exosomes increase the proliferation, migration, and invasion of normoxic CRC cells via miR-410-3p

**Figure 3:**
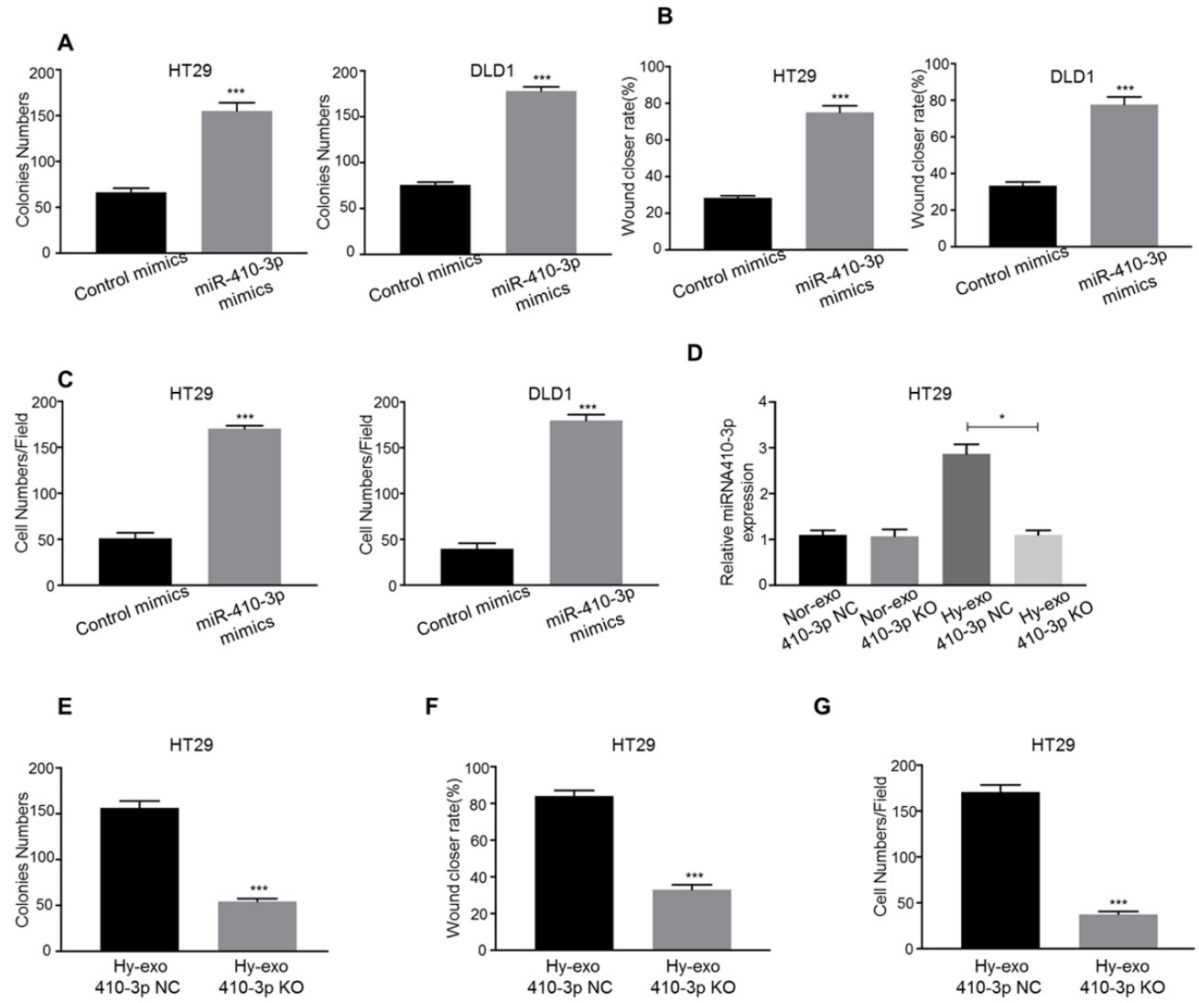
** Hypoxic CRC cells-secreted exosomes increase the proliferation, migration, and invasion of normoxic CRC cells via miR-410-3p.** (**A**) (**B**), and (**C**) CRC cells transfected with miR-410-3p mimics or miR-410-3p mimics NC were subjected to colony formation, wound healing, and transwell invasion assays. (**D**) Supernatants of hypoxic or normoxic HT29 cells transfected with miR-410-3p-knockout lentiviruses or miR-410-3p-knockout NC were collected for the isolation of exosomes. The level of mature miR-410-3p in normoxic CRC cells receiving these exosomes after a 12h treatment was assessed by quantitative real-time PCR. (**E**) (**F**), and (**G**) Normoxic HT29 cells treated with exosomes from hypoxic HT29 cells transfected with miR-410-3p-knockout lentiviruses or miR-410-3p-knockout NC were subjected to colony formation, wound healing, and transwell invasion assays. Error bars, SD. *P<0.05; ***P<0.001.

We explored the role of hypoxic exosome-derived miR-410-3p in the progression of CRC cells using miR-410-3p mimic transfection. Overexpression of miR-410-3p resulted in a significant increase in proliferation in CRC cells (Figure 3A). Consistent with above observations, both the migration and invasion abilities of CRC cells were significantly increased by transfected miR-410-3p mimics (Figure 3B, C).

Then, we isolated exosomes from the culture medium of hypoxic HT29 cells transfected with miR-410-3p-knockout lentivirus (410-3p KO). The knockout effect of miR-410-3p in exosomes was validated by RT-PCR. The expression of miR-410-3p was decreased by 2.6-fold in hypoxic HT29 cells-secreted exosomes (Figure 3D). We treated normoxic HT29 cells with hypoxic HT29 cells (410-3p KO)-secreted exosomes or hypoxic HT29 cells (410-3p NC)-secreted exosomes *in vitro*. As shown in Figure 3E, 410-3p NC-secreted exosomes effectively stimulated the proliferation, whereas 410-3p KO-secreted exosomes induced normoxic HT29 proliferation to a lesser extent than 410-3p NC. The result was confirmed by wound healing assay and transwell assay (Figure 3F, G).

### Hypoxic exosome-mediated transfer of miR-410-3p facilitates normoxic CRC cells progression by downregulating PTEN expression

**Figure 4:**
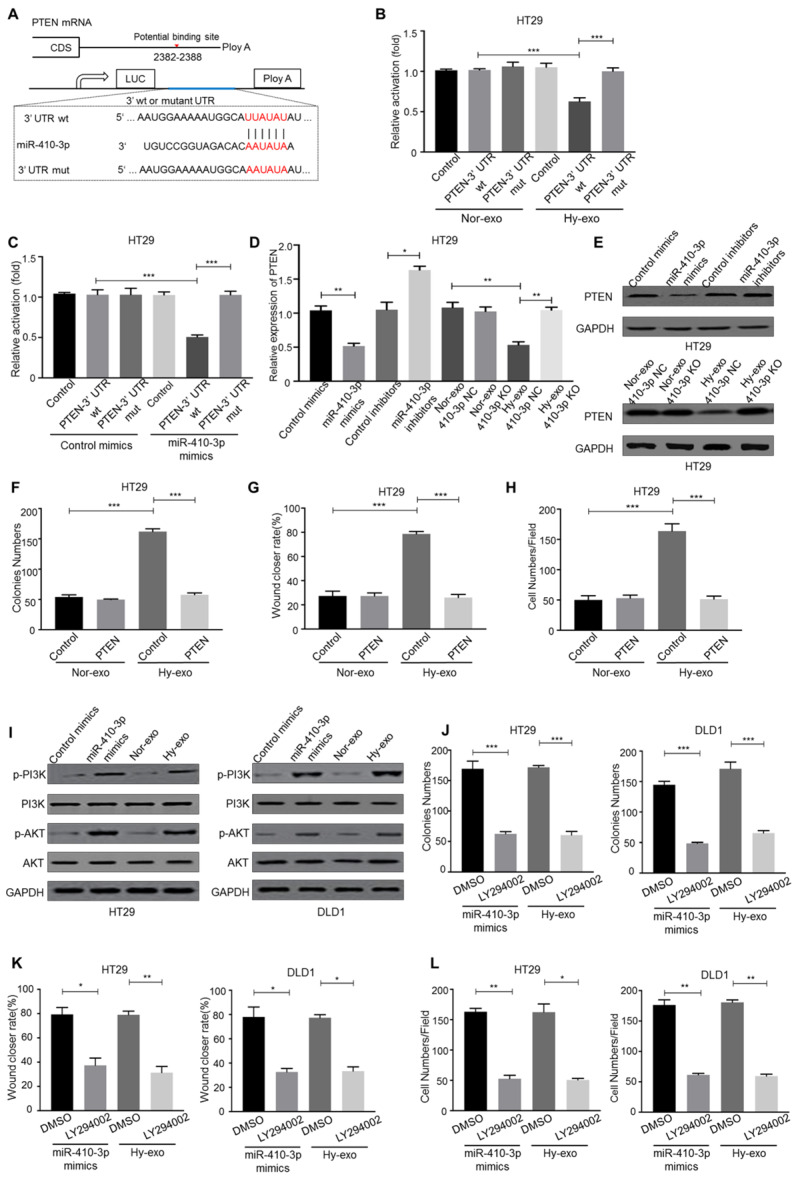
** Hypoxic exosome-mediated transfer of miR-410-3p facilitates normoxic CRC cells progression by downregulating PTEN expression.** (**A**) Schematic representation of the PTEN 3′UTR. Mutations were generated at the predicted miR-410-3p-binding sites. (**B-C**) The binding activity of hypoxic or normoxic HT29-derived exosomes and miR-410-3p mimics in the 3' UTR of PTEN, as determined by a 3' UTR luciferase report analysis. (**D**) MiR-410-3p mimics and hypoxic HT29-derived exosomes decreased the mRNA expression of PTEN in normoxic HT29 cells assessed by quantitative real-time PCR. (**E**) MiR-410-3p mimics and hypoxic HT29-derived exosomes decreased the protein expression of PTEN in normoxic HT29 cells assessed by Western blot. (**F**) (**G**), and (**H**) HT29 cells transfected with PTEN treated with exosomes from hypoxic or normoxic HT29 cells were subjected to colony formation, wound healing, and transwell invasion assays. (**I**) Hypoxic CRC-derived exosomes and miR-410-3p mimics increased the phosphorylation of PI3K and AKT assessed by Western blot. (**J-L**) PI3K inhibitors decreased the proliferation, migration, and invasion induced by hypoxic CRC-derived exosomes or miR-410-3p mimics assessed by colony formation, wound healing, and transwell invasion assays. Error bars, SD. *P<0.05; **P<0.01; ***P<0.001.

To deeply determine the targets of hypoxic exosome-derived miR-410-3p in the induction of CRC cells progression, we predicted its candidate target PTEN by three independent databases (TargetScan, miRanda and miRDB) (Figure 4A). Luc-PTEN-3' UTR luciferase reporter analysis has shown that hypoxic CRC cells-secreted exosomes and miR-410-3p mimics suppressed the luciferase activities of the PTEN 3′-UTR reporter constructs, whereas the effect was abolished when mutations were introduced into its seed sequences (Figure 4B, C). Moreover, hypoxic HT29 cells (410-3p NC)-secreted exosomes or miR-410-3p mimics significantly inhibited PTEN mRNA and protein levels (Figure 4D, E), while inhibition of miR-410-3p revealed the opposite effects (Figure 4D, E). Moreover, the effects of hypoxic HT29 cells derived exosomes on normoxic HT29 cells progression were also abolished by ectopic expression of PTEN (Figure 4F-H).

PTEN, as an inhibitor of the PI3K/Akt pathway, plays a critical role in tumor progression 25. Thus, we speculated hypoxic exosome-derived miR-410-3p could promote tumor progression through PI3K/Akt signaling pathway. And the phosphorylation level of PI3K and Akt in normoxic CRC cells treated with hypoxic exosomes or miR-410-3p mimics were determined and the expression level was higher than that in normoxic CRC cells treated with normoxic exosomes or miR-410-3p NC (Figure 4I). To investigate the role of PI3K/Akt signaling in hypoxic CRC cells-secreted exosomes-induced tumor progression, treatment of LY294002, a PI3K inhibitor, markedly blocked HT29 cells progression induced by miR-410-3p mimics and hypoxic HT29 cells-derived exosomes (Figure 4J-L).

### Hypoxic exosome-mediated transfer of miR-410-3p promotes metastasis of CRC cells *in vivo*

**Figure 5:**
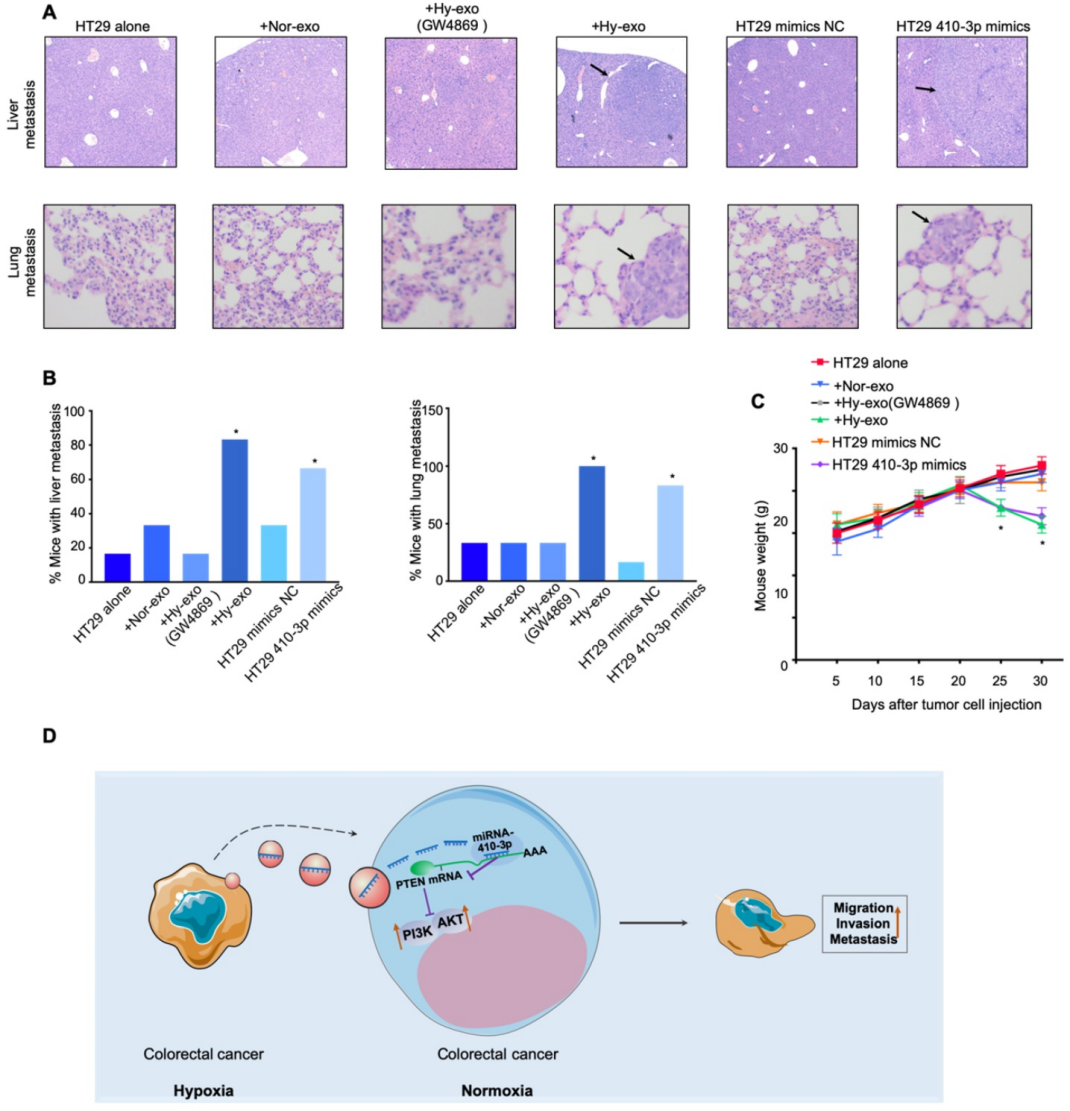
** Exosomes miR-410-3p promotes metastasis of CRC cells *in vivo*.** (**A**) Representative images of lung or liver metastasis in nude mice that resulted from HT29 cells alone or HT29 cells treated with hypoxic (with or without GW4869) or normoxic exosomes or HT29 cells transfected with miR-410-3p mimics/NC (**B**) Percentage of mice with metastasis is indicated from HT29 cells alone or HT29 cells treated with hypoxic (with or without GW4869) or normoxic exosomes or HT29 cells transfected with miR-410-3p mimics/NC (n=6 per group). (**C**) Weight of nude mice was monitored every 5 days after being injected with HT29 cells alone or HT29 cells treated with hypoxic (with or without GW4869) or normoxic exosomes or HT29 cells transfected with miR-410-3p mimics/NC via the tail veins. (**D**) Schematic model of hypoxic exosomal miR-410-3p promoting CRC progression. CRC cells-derived exosomal miR-410-3p under hypoxia mediates tumor progression via PTEN/PI3K/Akt signaling pathway, which facilitates the migration, invasion, and metastatic potential of CRC cells. Error bars, SD. *P<0.05.

To further assess the effect of hypoxic exosome-mediated transfer of miR-410-3p on tumor metastasis *in vivo*, HT29 cells stimulated by hypoxic HT29 cells-secreted exosomes (with or without GW4869) or stimulated by normoxic HT29 cells-secreted exosomes or transfected by miR-410-3p minics were injected into the mice via tail vein. Quantitation of the extent of metastasis by serial sectioning of the livers and lungs of mice in each group revealed livers metastatic lesions in 5 of 6 mice and lungs metastatic lesions in 6 of 6 mice in the HT29 cells stimulated by hypoxic HT29 cells-secreted exosomes (without GW4869) group (Figure 5A and B). Moreover, the weight of mice in the HT29 cells stimulated by hypoxic HT29 cells-secreted exosomes (without GW4869) group was significantly lower than control group (Figure 5C). Likewise, there were significantly more livers and lungs metastatic lesions, and lower weight in HT29 cells-miR-410-3p mimics group compared with control group (Figure 5A-C). These results demonstrate that hypoxia induced-exosomal miR-410-3p enhance the metastasis of CRC *in vivo*.

## Discussion

Hypoxia is a pivotal hallmark of various solid tumors and has a connection with tumor glycolysis, aggressive phenotypes, angiogenesis, and poor prognosis 26,27. Exosomal miRNAs have recently studied that are involved in the interactions between tumor cells and their surrounding microenvironment. As an important messenger between immune cells and cancer cells, exosomes can also be released from abundant non-malignant cells. Macrophages could transfer miR-365 to pancreatic adenocarcinoma cells through exosomes and induce drug resistance of these cancerous cells 28. Natural Killer-derived exosomal miR-186 could inhibit neuroblastoma growth and immune escape 29. Exosomes secreted by tumor cells can not only affect the surrounding normoxic tumor cells, but also can be transmitted to normal cells, such as mesenchymal stem cells (MSC) or primary macrophages, thereby promoting tumor progression 17,30. In this study, we extracted exosomes from the culture medium of hypoxic CRC cells and then investigated thoroughly the roles and mechanisms of exosomal miR-410-3p in the progression of normoxic CRC cells. We found that exosomal miR-410-3p derived from hypoxic CRC cells could increase the proliferation, migration, and invasion of normoxic CRC cells. Further study identified hypoxic CRC cells-secreted exosomes miR-410-3p facilitates an aggressive phenotype of CRC cells through PTEN/PI3K/Akt pathway (Figure 5D).

Hypoxia, a characteristic feature of tumor microenvironment, has emerged as a powerful driving force for tumor progression and resistance to therapy 26,31. Recently, there has been a large amount of evidence showing that exosomes are involved in tumor progression. However, the specific molecular mechanisms involved in the association between hypoxia and exosomes that regulate cancer progression have not been well elucidated. Recent studies have highlighted a critical role for hypoxic tumor cells in promoting the secretion of exosomes 32,33. Exosomes secreted from hypoxic tumor cells may induce tumor angiogenesis through modulating of endothelial cells 34. Furthermore, Jung and colleagues demonstrated that hypoxic exosome-mediated the reduction of cell-cell adhesion to promoted epidermoid carcinoma progression 35. Herein, we found that exosomes derived from hypoxic CRC cells promote tumor proliferation, migration and invasion of normoxic cells. Our results were in accordance with recent report showing that exosomes secreted from hypoxic oral squamous cell carcinoma cells are assimilated by normoxic cells and elicit recipient cells to a pro-metastatic phenotype 36. Similar to previous studies, our results revealed that important components of the tumor microenvironment include exosomes which have a role to exchange signals between cell-cell communication, and tumor cells in the hypoxic region could deliver exosomes to surrounding tumor cells in the normoxic region, making these recipient cells more aggressive and metastatic. The tumour microenvironment harbors multiple metabolic stressors including acidity and hypoxia, which have significant influences on remodeling tumour microenvironment. Recently, Logozzi et al reported that acidity both increase the number and reduce the size of the exosomes released by virtually all cancers 37. This is of paramount importance in as much as patients with cancer show increased levels of exosomes independently from their histology and propose a hypothesis that plasmatic levels of exosomes may represent a tumor biomarker, independently from their content 38,39. As far as acidity is concerned, a very recent paper has shown as culturing human cancer cells in acidic condition induce an increase of both expression and function of carbonic anhydrase IX on released exosomes 40. In summary, future anti-cancer strategies aimed at targeting both microenvironment acidity and hypoxia of tumour may be a viable approach.

Though recent studies have established the transfer of proteins between cells via exosomes in tumor progression, the functions and underlying mechanisms of non-coding RNAs molecules transportation via hypoxic exosomes have not been well clarified. Of note, it has been suggested that hypoxia microenvironment can affect the expression of miRNA in exosomes derived from tumor cells 36, and exosomal miRNAs have a critical function in regulating tumor progression. For instance, exosomal miR-25-3p from CRC cells promotes CRC progression by inducing the formation of a pre-metastatic niche, and it may be applied as a biomarker in diagnosis, prevention of cancer metastasis 41. Aberrant miR-410 expression has been observed in diverse types of cancers, thus playing different roles in cancer progression 42,43. For example, Qi et al. demonstrated miR-410 accelerated the proliferation and colony formation of lymphoblastic leukemia (ALL) cells through FKBP5 and Akt pathway 44. Zhang et al. showed that as an oncogene in NSCLC, miR-410 positively contribute to the tumorigenesis and development of NSCLC by down-regulating SLC34A2. In contrast, Xiong et al. demonstrated that miR-410-3p impaired gemcitabine resistance in pancreatic ductal adenocarcinoma by inhibiting HMGB1-mediated autophagy 23. Zhang et al. reported similar results, showing that miR-410-3p suppressed breast cancer progression by targeting Snail 45. Considering the discrepancy among different types of tumors, there may be tissue specific regulations of miR-410-3p in hypoxic microenvironment, and the function and mechanism of exosomal miR-410-3p in CRC progression is uncharacterized. As a known tumor suppressor, PTEN is frequently deactivated in various human cancers, including CRC 46-48. Growing studies have revealed that the inactivation of PTEN gene enhances the activation of PI3K/Akt and JAK2/STAT3 pathway, which in turn leads to the initiation, and malignant progression of cancers 49. Here, we have identified hypoxic CRC cells decrease PTEN expression by secreting exosomal miR-410-3p, and then the absence of PTEN in recipient normoxic cells is associated with the activation of PI3K and Akt. Understanding this specific molecular mechanism and then correlation to hypoxia and tumor progression will provide further incentives for targeting miR-410-3p as a potential strategy toward disrupting tumor microenvironment.

In conclusion, our study showed that hypoxia increased the miR-410-3p levels in CRC cells-derived exosomes. These miR-410-3p-rich CRC cells-derived exosomes were endocytosed by normoxic cells and promoted these normoxic cells progression and metastasis via PTEN/PI3K/Akt pathway. These findings highlighted that targeting exosomes or miR-410-3p might be a potential therapeutic target for combating CRC.

## Supplementary Material

Supplementary table S1.

## Figures and Tables

**Table 1:** Clinicopathologic characteristics of colorectal cancer patients

Characteristics	No. of patients
**Gender**	
Male	38
Female	22
**Age, years**	
<59	29
≥59	31
**Occurrence of liver metastases**	
Yes	21
No	39
**miR-410-3p expression**	
Low	29
High	31
**T stage**	
T1	1
T2	6
T3	38
T4	15
**Lymph node stage**	
N0	25
N1	18
N2	17
**M stage**	
M0	39
M1	21

## Abbreviations

CRC: Colorectal cancer
EMT: epithelial-mesenchymal transition
miRNA: MicroRNA
