# Supplementary material for: Exosomes Derived from Hypoxic Colorectal Cancer Cells Transfer miR-410-3p to Regulate Tumor Progression
Source: J Cancer. 2020 May 25;11(16):4724–35. doi: 10.7150/jca.33232 (PMC7330706; doi:10.7150/jca.33232)
Supplement: Supplementary file 1 — Supplementary table S1. [file jcav11p4724s1.pdf]

Supplementary Table 1. The sequences of the primers for quantitative RT-PCR

Gene	Primer Sequence (5′ to 3′)
PTEN	F: TTTGAAGACCATAACCCACCAC<br>R: ATTACACCAGTTCGTCCCTTTC
miR-410-3p	RT:GTCGTATCCAGTGCGTGTCGTGGAGTCGGCAATTGCACTGGATACGACACAGGC<br>F:GGGAATATAACACAGATG
Universal downstream	R: CAGTGCGTGTCGTGGAGT
GAPDH	F: GCACCACCAACTGCTTAGCA<br>R: GTCTTCTGGGTGGCAGTGATG
U6	F: CTCGCTTCGGCAGCACA<br>R: AACGCTTCACGAATTTGCGT
